# One-Step Preparation of Silk Fibroin and Sericin from Silkworm Cocoons Using High-Temperature High-Pressure Treatment

**DOI:** 10.3390/polym18151919

**Published:** 2026-08-05

**Authors:** Yeon Jin Kim, Bo Kyung Park, Ick Soo Kim, In Chul Um

**Affiliations:** 1 Department of Biofibers and Biomaterials Science, Kyungpook National University, Daegu 41566, Republic of Korea; kyj60621@naver.com (Y.J.K.); qhrid1004@naver.com (B.K.P.); 2 Institute for Fiber Engineering and Science (IFES), Research Cluster for Social Implementation, Shinshu University, Nagano 386-8567, Japan; kim@shinshu-u.ac.jp

**Keywords:** fibroin, sericin, HTHP method, simultaneous preparation, silk biomaterials

## Abstract

Conventional silk degumming processes are primarily designed to recover either silk fibroin (SF) or sericin, resulting in inefficient utilization of silk resources and substantial sericin waste. In this study, a one-step high-temperature high-pressure (HTHP) process was developed for the simultaneous preparation of SF and sericin from silkworm cocoons. To determine the optimum processing conditions, HTHP treatment was performed at 120 °C for 10, 20, and 30 min. The effects of treatment time on the degumming efficiency and the physicochemical properties of SF and sericin were systematically investigated through the characterization of SF as degummed fibers, regenerated solutions, and electrospun fibers, and sericin as solutions and gels. Complete degumming was achieved after 10 min of HTHP treatment, corresponding to a degumming ratio of 25.4%, with no further increase at longer treatment times. While complete degumming was maintained beyond 10 min, prolonged treatment progressively deteriorated the physicochemical properties of sericin and eventually reduced the molecular integrity of SF, as consistently demonstrated across the different material forms. Compared with the conventional soap/soda method, the HTHP process better preserved the molecular integrity of SF while simultaneously recovering an aqueous sericin fraction without chemical degumming. Based on the degumming efficiency and the physicochemical properties of both silk proteins, 10 min was identified as the optimum HTHP treatment time for the simultaneous preparation of SF and sericin. This one-step HTHP process provides a simple and efficient process for improving silk resource utilization and facilitating the industrial production of SF and sericin-based biomaterials.

## 1. Introduction

Silk is a composite fiber composed of two biopolymers: fibroin and sericin. Silk fibroin (SF) shows excellent blood compatibility [1,2], good cytocompatibility [3,4], and biodegradability [5,6]. With its useful properties as a biomaterial, SF has been intensively studied for biomedical applications including membranes for guided bone regeneration [7,8], tissue engineering scaffolds [9], wound dressing [10], bone substitutes [11], artificial heart valves [12], nerve conduits [13], and tympanic membranes [14,15].

Silk sericin is removed from silk using a degumming process to improve the luster and tactile feeling of silk. Traditionally, sericin has been regarded as a waste byproduct and discarded during silk processing. Recently, it was reported that silk sericin has antioxidant effects [16], antibacterial properties [17], ultraviolet light blocking effects [18], moisturizing effects on the skin [19], cholesterol-lowering effects [20,21], and wound-healing effects [22,23]. Therefore, sericin is mainly used in biomedical and cosmetic applications [24,25,26].

For these application studies of SF and sericin, they are extracted from silk fibers using various preparation methods. Conventional silk degumming is generally carried out by treating silk fibers in boiling aqueous media, either with water alone or, more commonly, with chemical additives such as soap and sodium carbonate to improve sericin removal efficiency. That is, the preparation methods for SF include the urea method, high-temperature high-pressure (HTHP) method, acid method, soap/soda method, soda method [27], enzymatic degumming [28], steam degumming [29], and Na_2_CO_3_ method [30]. The extraction methods for sericin include the HTHP method [31,32,33], the citric acid method, the sodium carbonate method, the urea method [31,32], the infrared (IR) heating method [34], and the NaCl method [35].

These degumming processes effectively remove sericin from raw silk fibers, yielding degummed SF fibers suitable for subsequent processing and applications. However, because many degumming methods employ chemical reagents, sericin remains in the degumming solution together with residual chemicals, requiring an additional reagent-removal step, such as dialysis or desalting, to obtain high-purity sericin. These downstream processes increase processing complexity and production costs, making sericin recovery economically less attractive. Consequently, despite its valuable properties and wide range of potential applications, sericin is frequently discarded as an industrial byproduct. As a result, approximately 50,000 tons of sericin are discarded annually, leading to substantial resource loss and potential environmental burdens [36,37]. This challenge is exemplified by the work of Han et al., in which sericin extracted using an aqueous Na_2_CO_3_ solution required subsequent dialysis to remove residual chemicals, while the degummed SF fibers underwent repeated washing and drying before further use [38]. Consequently, such additional processing requirements hinder the efficient utilization of sericin and limit the large-scale industrial production of SF- and sericin-based biomaterials.

Although HTHP treatment has been previously used for silk degumming and sericin extraction, most studies have evaluated either the degummed SF or the extracted sericin as the primary product. For example, Zhao and Zhang [39] investigated a greener degumming process to recover layered sericin peptides and evaluated their physicochemical characteristics and bioactivities. However, their primary focus was the recovery and characterization of sericin rather than the simultaneous utilization of both silk protein fractions. Consequently, limited information is available on the processing window that simultaneously ensures sufficient sericin removal, preservation of SF integrity, and recovery of sericin with relatively limited molecular degradation.

In this study, HTHP treatment was not proposed as a fundamentally new degumming technique. Instead, the treatment time was systematically optimized for the simultaneous recovery of SF and sericin by evaluating the effects of processing conditions on both protein fractions. SF was characterized as degummed fibers, regenerated solutions, and electrospun fibers, whereas sericin was evaluated in solution, powder, and gel forms. This integrated approach was used to determine processing conditions that provided effective separation while limiting the deterioration of both silk proteins. This strategy is expected to improve the efficiency of silk resource utilization and provide a basis for developing a simplified simultaneous recovery process for SF- and sericin-based materials.

## 2. Experimental Section

### 2.1. Materials

To extract fibroin and sericin, Baekokjam silk cocoons (*Bombyx mori*) were used. The silkworm cocoons were provided by the National Institute of Agricultural Science (Wanju, Republic of Korea). Sodium oleate (extra pure, Junsei Chemical Co., Ltd., Tokyo, Japan), sodium carbonate (anhydrous, assay ≥ 99.0%, OCI Co., Ltd., Seoul, Republic of Korea), calcium chloride (anhydrous, practical grade, Duksan Pure Chemicals Co., Ltd., Ansan, Republic of Korea), and ethanol (absolute, for analysis, Sigma-Aldrich, Darmstadt, Germany) were used as received.

### 2.2. Separation of SF and Sericin

First, nonprotein components in the silk cocoons were removed by soaking the silk cocoons in a 70% (*v*/*v*) aqueous ethanol solution at 50 °C for two days. The ratio of the cocoons to the aqueous ethanol solution was 1:30 (*w*/*v*). The silkworm cocoons were then washed with purified water and finally dried at room temperature [40].

**Figure 1:**
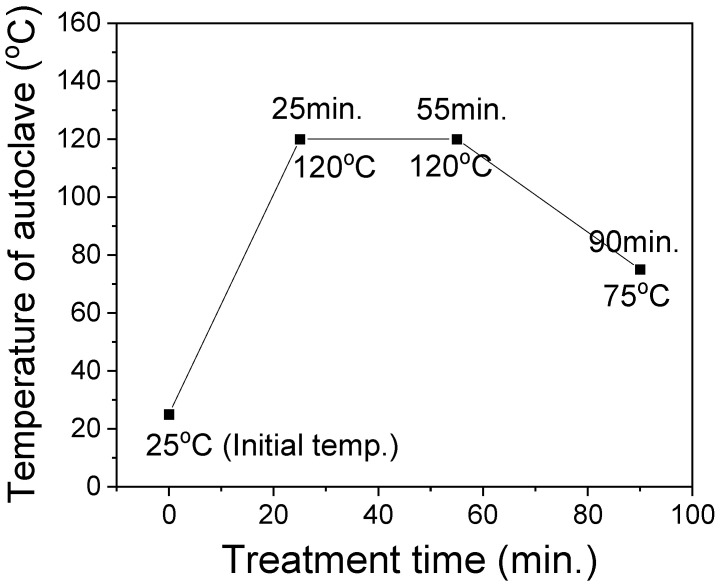
Temperature profile of the high-temperature high-pressure (HTHP) treatment used for degumming silkworm cocoons. The representative treatment conditions shown correspond to 30 min at 120 °C.

Then, the separation of SF and sericin from the silkworm cocoons was conducted using degumming processes. In this study, two separation methods were used to obtain SF and silk sericin: the HTHP method and the soap/soda method. Figure 1 shows the treatment temperature and time conditions during the treatment of silk using the HTHP method. The silkworm cocoons were immersed in purified water in an autoclave (JSAT-65, JSR, Gongju, Republic of Korea). The bath temperature in the autoclave was raised to 120 °C over 25 min and then maintained at 120 °C for 5, 10, 20, and 30 min. The SF samples prepared under these conditions were named HTHP05SF, HTHP10SF, HTHP20SF, and HTHP30SF, respectively. Similarly, the sericin samples prepared under these conditions were named HTHP10SS, HTHP20SS, and HTHP30SS, respectively. After treatment, the samples were cooled to 75 °C for 35 min. Finally, the degummed silk was separated from the aqueous sericin solution by filtration through a polyester nonwoven fabric and then washed with purified water. The purified water was obtained using a water purification system (RO50, Hana Science, Hanam, Republic of Korea) equipped with a reverse-osmosis membrane. The ratio of the cocoons to purified water in the bath was 1:50 (*w*/*v*).

For the soap/soda method, the silk cocoons were degummed in a boiling aqueous solution containing 0.3% (*w*/*v*) sodium oleate and 0.2% (*w*/*v*) sodium carbonate at 100 °C for 1 h. The ratio of the cocoons to purified water was 1:25 (*w*/*v*). The degummed silk (SF) was named SPSD60SF. After the separation process, aqueous sericin solution and SF were obtained. SF was washed with purified water and dried at 105 °C for 24 h. The aqueous sericin solution was filtered using nonwoven polyester fabrics and dried to obtain silk sericin powder. For reference, the soap/soda treatment was used as a conventional reference method rather than as a parameter-matched control. Because the two processes differed in bath ratio, chemical composition, temperature, treatment time, and thermal history, the comparison was intended to benchmark the overall outcomes of the respective processing protocols and not to isolate the independent effects of pressure, temperature, or treatment medium.

### 2.3. Preparation of the Sericin Solution and Gel

The silk sericin powder was dissolved in 98% formic acid at 55 °C for 30 min to prepare 0.3% (*w*/*w*) and 1.5% (*w*/*w*) silk sericin formic acid solutions for viscosity and gel strength measurements, respectively. Formic acid was selected because it effectively dissolved sericin while minimizing gelation during rheological measurements. The 1.5% (*w*/*w*) silk sericin formic acid solutions with different HTHP extraction times were stored at 4 °C for three days to fabricate sericin gels.

### 2.4. Preparation and Electrospinning of the Regenerated SF

The regenerated SF was prepared using a previously described method [41,42]. Briefly, SF was dissolved in a ternary solvent containing CaCl_2_/H_2_O/EtOH (1/8/2 molar ratio) at 85 °C for 30 min. The ratio of SF to the solvent was 1:20 (*w*/*v*). The aqueous regenerated SF solution was obtained by dialyzing the dissolved solution in a cellulose tube (MW cutoff = 12,000–14,000 Da) against water for four days at room temperature. The aqueous SF solution was filtered and dried to obtain regenerated SF powder.

The detailed procedure for the electrospinning of the regenerated SF solution has been described previously [5]. The regenerated SF was dissolved in 98% formic acid and filtered using nonwoven polyester fabrics to prepare a dope solution for electrospinning. A 7% (*w*/*w*) SF formic acid solution was loaded into a plastic syringe fitted with a 21-gauge stainless steel needle (inner diameter = 0.337 mm) at the tip. The electrospinning process was performed at a voltage of 20 kV, and the tip-to-collector distance was fixed at 10 cm.

### 2.5. Measurement and Characterization

The following equation was used to calculate the degumming ratios [43,44]:

Degumming ratio (%) = (1 − dry weight of degummed cocoons/dry weight of native cocoons) × 100.

The dry weight of each cocoon was measured using a moisture analyzer (XM60, Precisa Gravimetrics, Dietikon, Switzerland).

The morphologies of the silkworm cocoons, SF, and electrospun SF fibers were examined using scanning electron microscopy (SEM; S-4800, Hitachi, Tokyo, Japan). The silk samples were coated with Pt–Pd before observation. The mean and standard deviation of the diameter of the electrospun SF fibers were obtained by measuring 50 SF fibers from SEM images using an image analysis program (DIMIS-PRO 2.0, Siwon Optical Technology, Anyang, Republic of Korea).

The molecular conformation of the silkworm cocoons and SF was examined using Fourier-transform infrared (FTIR) spectroscopy (Nicolet 380, Thermo Fisher Scientific, Waltham, MA, USA) with the attenuated total reflection (Smart iTR ZnSe) method. The scan range, number of scans, and resolution were 650–4000 cm^−1^, 32, and 8 cm^−1^, respectively. The crystallinity index was calculated from the FTIR spectrum using the following equation [40,44]:

$$\left.Crystallinity\;index\;(\%\right)\;=\;\frac{A_{1260{cm}^{-1}}}{A_{1230{cm}^{-1}}}\;\times \;100$$

where A_1230cm^−1^_ is the absorbance at 1230 cm^−1^ (attributed to random coil conformation related to the amorphous region) and A_1260cm^−1^_ is the absorbance at 1260 cm^−1^ (due to *β*-sheet crystallite related to the crystalline region). The mean and standard deviation of the crystallinity index were obtained from the seven FTIR measurements.

For the rheological measurements, 10% (*w*/*w*) regenerated SF formic acid solution and 0.3% (*w*/*w*) sericin formic acid solution were used. The shear viscosity was measured by a rheometer (HAAKE MARS III, Thermo Fisher Scientific, Karlsruhe, Germany) using a cone and plate geometry with a shear rate of 0.1–100 s^−1^ at 25 °C. The radius and angle of the cone were 60 mm and 1°, respectively [45,46].

Differential scanning calorimetry (DSC) analysis was performed using a thermal analysis instrument (DSC 25, TA Instruments, New Castle, DE, USA) in the range of 60–270 °C at a scan rate of 10 °C/min under a nitrogen gas flow rate of 50 mL/min.

The gelation behavior of sericin was examined via an axial test using a rheometer (HAAKE MARS III, Thermo Fisher Scientific, Karlsruhe, Germany) with a 35-mm parallel-plate geometry at 25 °C [28]. The 35-mm plate of the rheometer compressed the sericin samples at a speed of 0.2 mm/s. The compression strength obtained from the axial test was used as the gel strength (Pa) of the sericin samples [47].

All measurements were conducted at least in triplicate, and the results are presented as mean ± standard deviation.

## 3. Results and Discussion

### 3.1. Structural Characteristics of SF

**Figure 2:**
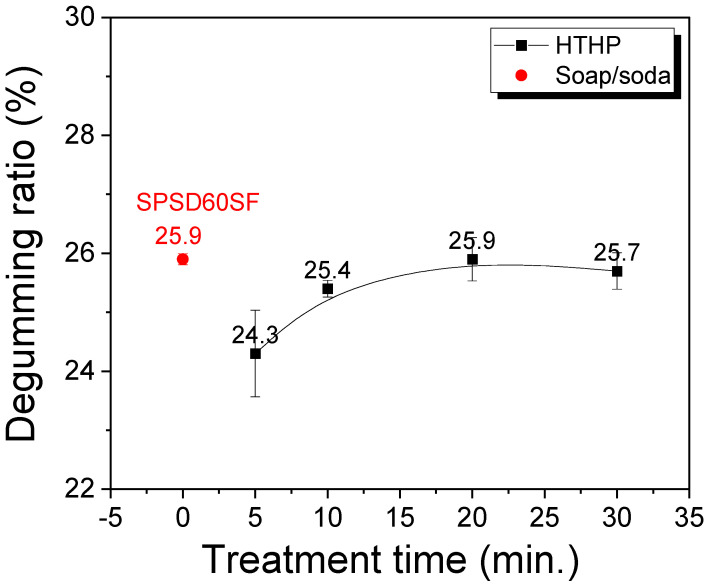
Degumming ratio of silk samples prepared by the soap/soda and HTHP methods. Error bars represent the standard deviation (*n* = 3).

The silkworm cocoons were degummed at different treatment times using the HTHP method to separate SF and sericin from the cocoons, and the degumming ratio of the silkworm cocoons is shown in Figure 2. As the treatment time at 120 °C increased to 10 min, the degumming ratio increased to 25.4% and did not increase significantly thereafter. The silkworm cocoons were also degummed using the soap/soda method for 60 min as a conventional reference treatment for extensive degumming, resulting in a degumming ratio of 25.9%. This treatment was used as a benchmark rather than as a parameter-matched control because the two processes differed in treatment medium, bath ratio, temperature, duration, and thermal history. The value obtained by the soap/soda method was regarded as an approximate reference for the removable sericin fraction of the cocoons [27,48]. The comparable degumming ratios suggest that substantial sericin removal was achieved after at least 10 min of HTHP treatment at 120 °C.

**Figure 3:**
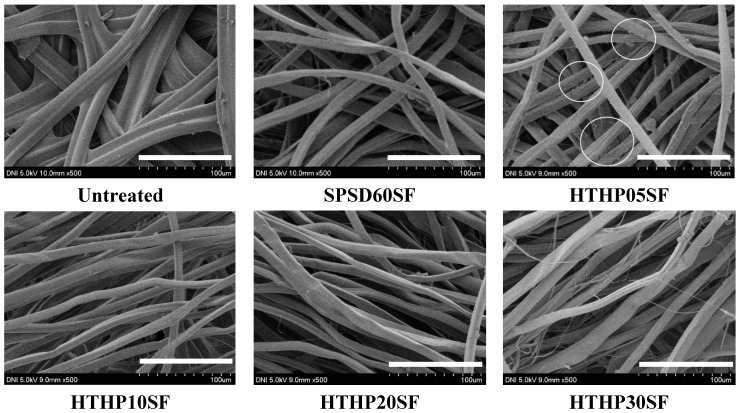
FE-SEM images of untreated silkworm cocoons and silk samples degummed by the soap/soda and HTHP methods. The scale bar represents 100 µm. The white circles in the HTHP05SF image indicate regions where a small amount of residual sericin is observed.

SEM measurement was conducted to examine whether sericin was removed from the silkworm cocoons, and the results are shown in Figure 3. In the case of untreated cocoons (i.e., raw cocoons), sericin covered the two SF strands. For SPSD60SF, sericin was not observed on the SF surface, whereas SF nanofibrils were clearly detected. The SF nanofibrils were generated when the covering material (sericin) was fully removed and the SF fibers slightly damaged [27,49,50]. Therefore, the presence of SF nanofibrils is considered evidence that sericin was removed from the silkworm cocoons.

In the case of HTHP05SF, the surface of the SF fibers was not smooth, and a small amount of residual sericin was still observed on the SF fibers, as indicated by the white circles in the figure. This result is consistent with the relatively low degumming ratio of HTHP05SF (24.3%) shown in Figure 2. For HTHP10SF and HTHP20SF, smooth and clean SF fiber surfaces were observed, indicating that sericin was completely removed in these samples. In the case of HTHP30SF, the SF fibers showed both smooth fiber surfaces and SF nanofibrils, indicating that sericin was completely removed and that the SF fibers were slightly damaged during the degumming process. These SEM results of HTHP10SF, HTHP20SF, and HTHP30SF agree well with the degumming ratio results (25.4–25.9%) shown in Figure 2. Unlike HTHP10SF and HTHP20SF, the presence of SF nanofibrils in HTHP30SF implies that HTHP30SF experienced greater structural damage during prolonged HTHP treatment.

Based on the results in Figure 2 and Figure 3, a treatment time of 10–30 min under HTHP conditions is appropriate to obtain SF and sericin from the silkworm cocoons at the same time. Because HTHP05SF contained silk sericin, it is not appropriate for finding the optimum condition for preparing both SF and sericin from silkworm cocoons. Therefore, HTHP05SF was excluded from further examination. Also, we did not treat the cocoons for more than 30 min under HTHP conditions because prolonged degumming treatment may cause hydrolytic degradation of sericin [51,52] and SF [27,49]. Consequently, HTHP10SF, HTHP20SF, and HTHP30SF were used in the subsequent examinations.

**Figure 4:**
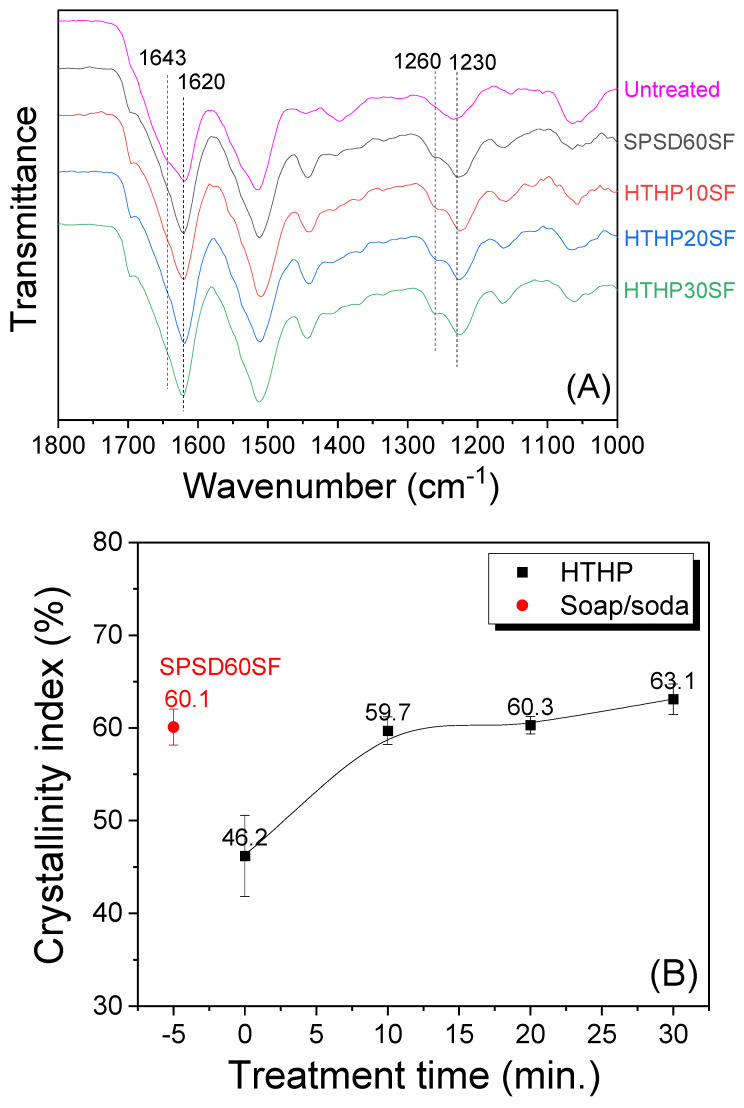
(**A**) ATR-FTIR spectra of untreated silkworm cocoons and silk samples degummed by the soap/soda and HTHP methods. (**B**) Crystallinity index of untreated cocoons and degummed silk samples. Error bars represent the standard deviation (*n* = 7).

The molecular conformation and crystallinity of silk strongly affect its mechanical properties [48,53]. FTIR measurements have been extensively conducted to examine the molecular conformation of silk materials [46,53,54,55]. To examine the effect of degumming time in the HTHP method on the molecular conformation of silk, FTIR measurements were performed on the degummed silk samples, and the results are shown in Figure 4A. The untreated cocoons showed an IR absorption peak at 1620 cm^−1^ and a shoulder peak at 1643 cm^−1^ in the amide I band attributed to the β-sheet crystallite and random coil conformations, respectively, and an IR peak at 1230 cm^−1^ attributed to random coil conformation. This indicates that the β-sheet crystallite and random coil conformations coexist in the raw cocoons. In the degummed silk samples (SPSD60SF, HTHP10SF, HTHP20SF, and HTHP30SF), the IR absorption peaks at 1620 and 1260 cm^−1^ became stronger, and the shoulder peak at 1643 cm^−1^ disappeared, indicating that the silk became more crystallized after the degumming treatment, which is consistent with previous reports [27,50].

To quantitatively examine the change in the crystallinity of silk, the crystallinity index was calculated from the FTIR spectra, and the results are shown in Figure 4B. The crystallinity index is widely used as a simple indicator of silk crystallinity and shows good correlation with the molecular conformations obtained from FTIR spectral deconvolution [56]. As the treatment time in the HTHP method increased to 10 min (HTHP10SF), the crystallinity of silk significantly increased from 46.2% to 59.7% and was maintained until 20 min (HTHP20SF). HTHP30SF showed a higher crystallinity index than HTHP10SF and HTHP20SF.

The increase in the crystallinity index up to 10 min is attributed to the removal of sericin, which has lower crystallinity than SF [27,50]. The slight increase in crystallinity at 30 min is attributed to the removal of the less crystallized part of SF during over-degumming. Lee et al. [50] reported that the less crystallized interfibrillar structure of SF is removed by over-degumming, resulting in an increase in the crystallinity index of silk and the generation of silk nanofibrils. Similarly, in this study, both the increased crystallinity index (Figure 4B) and the generation of nanofibrils on SF (Figure 3) were observed at a treatment time of 30 min, supporting the occurrence of partial structural degradation during prolonged HTHP treatment.

### 3.2. Rheological Properties and Electrospinnability of the Regenerated SF Solutions

Although the 10 to 30 min treatment time under HTHP conditions was appropriate for obtaining SF and sericin at the same time, partial molecular degradation of SF and sericin may occur during treatment under HTHP conditions, resulting in a decrease in MW. That is, it is important to find the optimum conditions to obtain SF and sericin with relatively preserved molecular structure.

**Figure 5:**
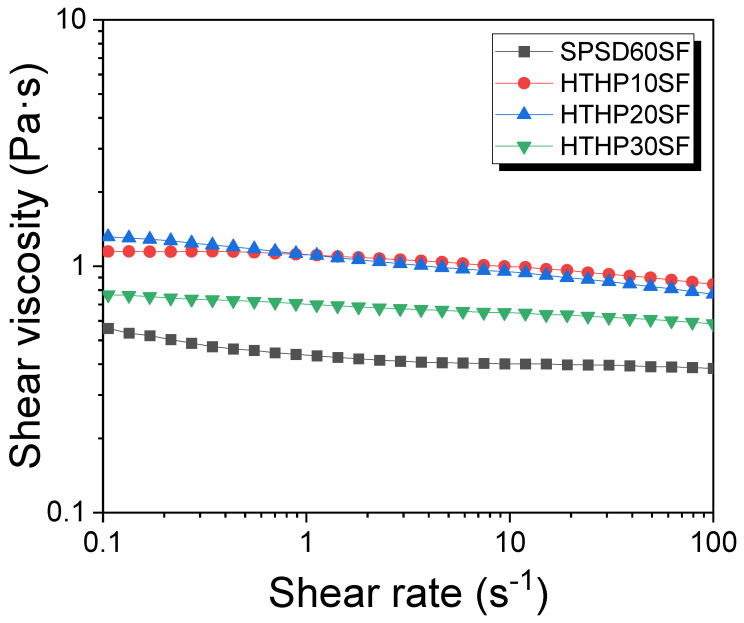
Steady-state flow curves of 10% (*w*/*w*) regenerated SF formic acid solutions prepared by the soap/soda and HTHP methods.

SDS-PAGE [57], liquid chromatography [42,51,58,59], and viscosity measurement using a rheometer [27,42,58] have been used to evaluate the MW of silk polymers. Among them, viscosity measurement using a rheometer is the simplest and most convenient method to compare (i.e., evaluate relatively) the molecular state (MW) of silk samples. Therefore, we conducted steady-state flow measurements for 10% silk solutions, and the results are shown in Figure 5. Although a very slight shear-thinning was shown in all the samples, all SF formic acid solutions showed almost Newtonian fluid behavior, which is consistent with the results of the SF formic acid solutions reported previously [42,45,60].

The HTHP10SF and HTHP20SF solutions showed very similar viscosities. However, HTHP30SF showed lower viscosities than HTHP10SF and HTHP20SF, indicating greater molecular degradation of SF during prolonged HTHP treatment. As shown in Figure 3, increasing the HTHP treatment time caused structural damage to SF fibers, resulting in the generation of nanofibrils on the SF surface and partial molecular degradation of SF [27,49].

SPSD60SF showed the lowest viscosity among the SF samples tested. Kim et al. [27] reported that the viscosity of the SF solution obtained under HTHP conditions is higher than that obtained using the soap/soda degumming method. Therefore, the results in Figure 5 are consistent with the findings of the previous report [27]. These results indicate that the SF samples obtained under HTHP conditions used in this study have a higher MW than the SF obtained using the soap/soda degumming method.

**Figure 6:**
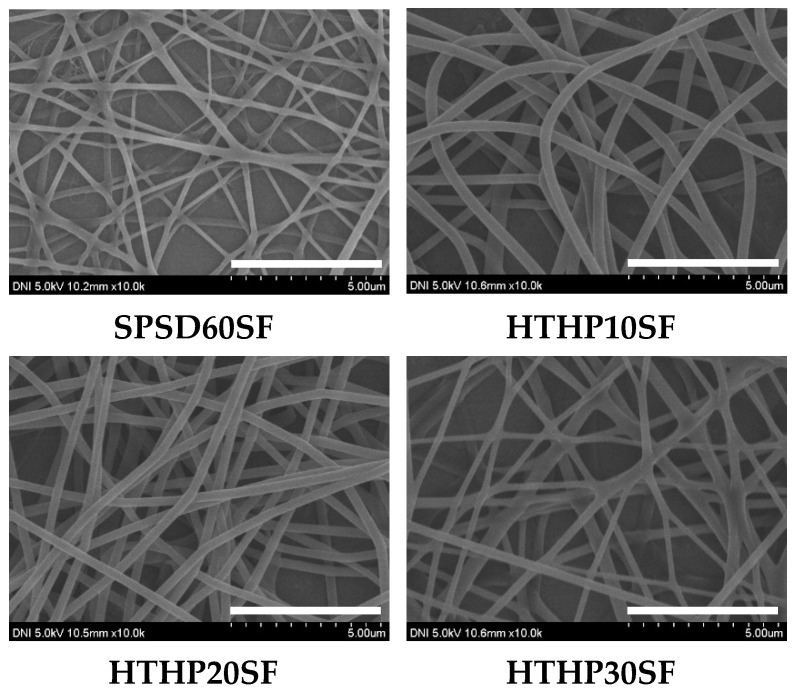
FE-SEM images of electrospun SF fibers prepared from silk degummed by the soap/soda and HTHP methods. The scale bar represents 5 µm.

**Figure 7:**
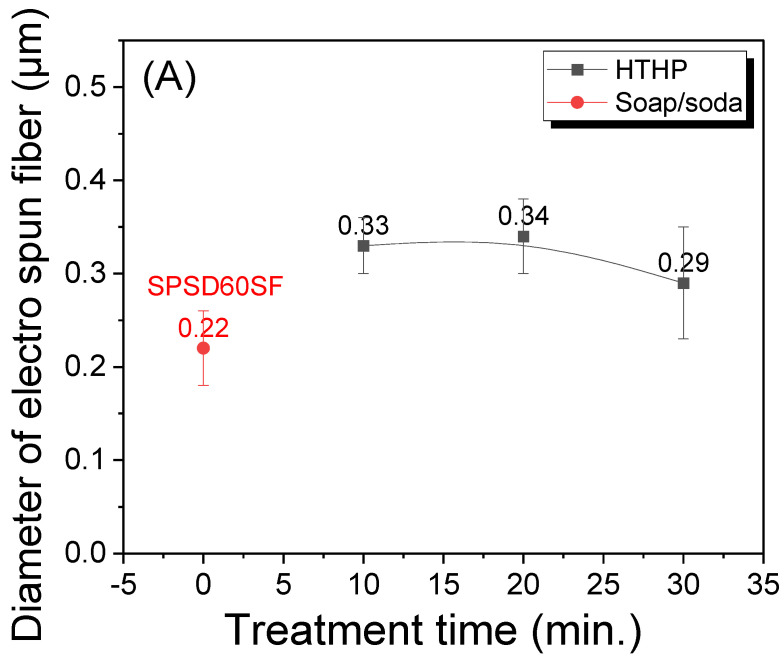

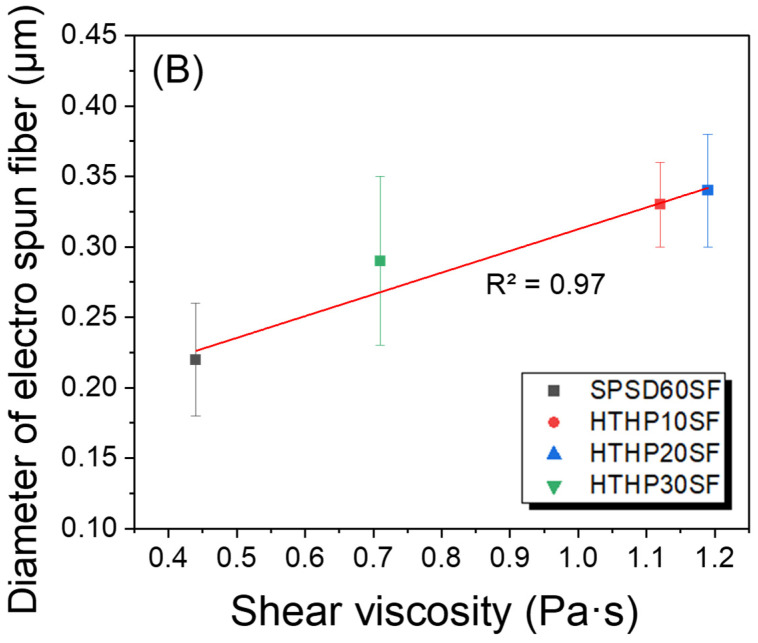
(**A**) Diameter of electrospun SF fibers prepared by the soap/soda and HTHP methods (*n* = 50). (**B**) Correlation between shear viscosity and electrospun fiber diameter.

To confirm the validity of the viscosity results in Figure 5, we conducted electrospinning of the SF solution because the diameter of the electrospun SF fibers shows a good correlation with the viscosity of the SF solution [45,49,61,62]. That is, a measurement of the diameter of the electrospun SF fibers can be used as another tool to compare the MWs of SF. We performed the electrospinning of the SF solutions, and the results are exhibited in Figure 6. All the SF solutions showed good electrospinnability, resulting in good fiber formation. However, the diameters of the electrospun SF fibers were different depending on the SF samples. Therefore, the diameter of the electrospun SF fibers was measured from the SEM images, and the results are shown in Figure 7A.

HTHP10SF and HTHP20SF showed similar diameters (0.33–0.34 µm). HTHP30SF showed a lower diameter (0.29 µm) than HTHP10SF and HTHP20SF. SPSD60SF displayed the lowest diameter among the samples (0.22 µm). Figure 7 shows a good correlation between the viscosity of the SF solution and the diameter of the electrospun SF fibers (*R*^2^ = 0.97), supporting the close relationship between solution viscosity and electrospun fiber diameter reported in previous studies [45,49,61,62]. Also, this good correlation between them reconfirms the reliability of the results of the viscosity measurement in Figure 5. Based on the results of the viscosity of the SF solution and the diameter of the electrospun SF fibers, HTHP10SF and HTHP20SF are the optimum samples for obtaining SF with relatively preserved molecular integrity.

### 3.3. Rheological Properties, Thermal Stability, and Gelation of Silk Sericin

**Figure 8:**
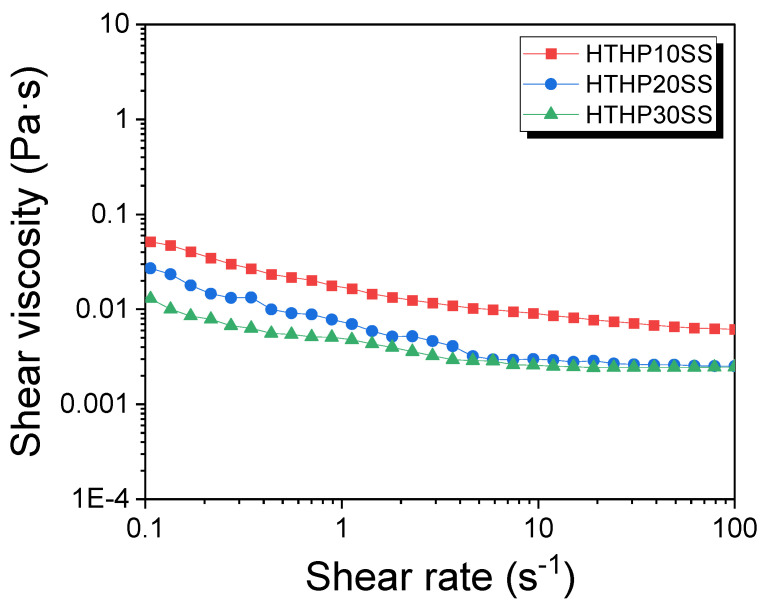
Steady-state flow curves of 0.3% (*w*/*w*) silk sericin formic acid solutions prepared at different treatment times under HTHP conditions.

A steady-state flow test was conducted to examine the rheological properties of the silk sericin formic acid solution to indirectly evaluate the MW of sericin prepared under different treatment times in the HTHP method, and the results are shown in Figure 8. All the sericin formic acid solutions showed shear-thinning in the steady-state flow test, which is consistent with the results in a previous report [52]. In the case of HTHP10SS, the shear viscosity at 0.1 s^−1^ was 50 mPa∙s. However, as the treatment time increased to 30 min (i.e., HTHP30SS), the shear viscosity at 0.1 s^−1^ decreased to 10 mPa∙s. This indicates that the MW of silk sericin decreased with increasing treatment time due to the hydrolytic degradation of sericin under HTHP conditions [51,52,59].

**Figure 9:**
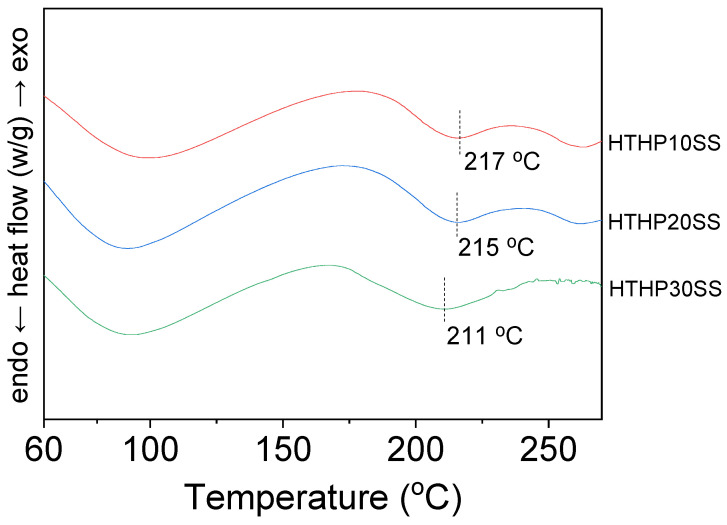
DSC thermograms of silk sericin powders prepared at different treatment times under HTHP conditions.

The thermal stability of silk sericin can be evaluated by measuring its thermal decomposition temperature and is influenced by its MW [28,63]. Therefore, the thermal decomposition temperature of sericin was measured using DSC to confirm the evaluation of its MW via the solution viscosity (Figure 8), and the results are shown in Figure 9.

HTHP10SS showed a broad endothermic peak at 217 °C, which can be attributed to the thermal degradation of sericin [28,63]. As the treatment time increased to 30 min (HTHP30SS), the peak shifted to a lower temperature (211 °C), which indicates that the MW of sericin decreased with increasing treatment time under HTHP conditions, confirming the results of the rheological properties of sericin (Figure 8).

**Figure 10:**
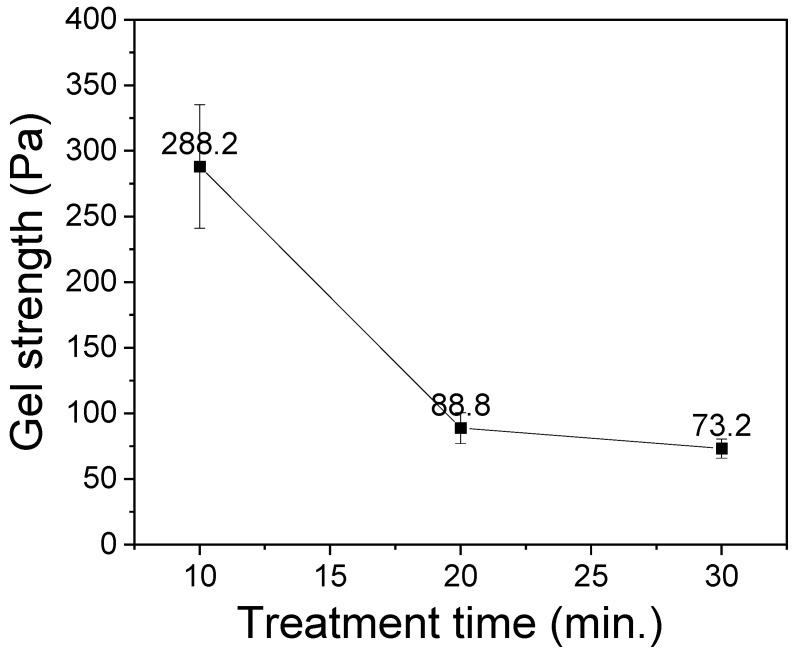
Effect of HTHP treatment time on the gel strength of silk sericin (*n* = 6).

A sericin solution easily becomes a gel [47,51], and the gel strength of sericin shows a positive correlation with its MW [51]. Therefore, the gel strength of the sericin gel prepared from the 1.5% sericin solution was measured via an axial test using a rheometer, and the results are displayed in Figure 10. HTHP10SS showed a gel strength of 288 Pa, which decreased to 73 Pa as the treatment time increased to 30 min (HTHP30SS), implying that the MW of sericin decreased with increasing treatment time, reconfirming the results of viscosity and thermal decomposition temperature.

Based on the results above, in the case of SF, treatment times of 10 and 20 min are appropriate for obtaining the highest MW of SF. For sericin, a treatment time of 10 min is the best to obtain sericin with minimal molecular degradation. Conclusively, a treatment time of 10 min under HTHP conditions was determined to be the optimum condition for the simultaneous preparation of SF and sericin with relatively preserved molecular structures from silkworm cocoons.

## 4. Conclusions

This study examined the structural characteristics and properties of SF and sericin prepared at different treatment times under HTHP conditions to determine the optimum condition for the simultaneous preparation of SF and sericin with relatively preserved molecular integrity. The degumming ratio results indicate that treatment for more than 10 min under HTHP conditions is required to effectively separate SF and sericin from silkworm cocoons. The results of the viscosity of the SF solution and the diameter of the electrospun SF fibers showed that treatment times of 10 and 20 min were the optimum conditions for preparing SF with minimal molecular degradation. In addition, the viscosity, thermal decomposition temperature, and gel strength results of sericin indicated that a treatment time of 10 min was the optimum condition for obtaining sericin with the most preserved molecular structure.

Conclusively, 10 min was determined to be the optimum treatment time under HTHP conditions for the simultaneous preparation of SF and sericin with relatively preserved molecular structures from silkworm cocoons. The results of this study provide useful information for the efficient utilization of silk resources. Furthermore, the proposed HTHP process may contribute to the simultaneous valorization of SF and sericin while reducing sericin waste during silk processing.

## Data Availability

The original contributions presented in this study are included in the article. Further inquiries can be directed to the corresponding author.
